# Multi-Additivity in Kaniadakis Entropy

**DOI:** 10.3390/e26010077

**Published:** 2024-01-17

**Authors:** Antonio M. Scarfone, Tatsuaki Wada

**Affiliations:** 1 Istituto dei Sistemi Complessi—Consiglio Nazionale delle Ricerche (ISC-CNR), c/o Dipartimento di Scienza Applicata e Tecnologia del Politecnico di Torino, Corso Duca degli Abruzzi 24, 10129 Torino, Italy; 2 Region of Electrical and Electronic Systems Engineering, Ibaraki University, 4-12-1 Nakanarusawa-cho, Hitachi 316-8511, Ibaraki, Japan; tatsuaki.wada.to@vc.ibaraki.ac.jp

**Keywords:** *κ*-entropy, pseudo-additivity, power-law distributions

## Abstract

It is known that Kaniadakis entropy, a generalization of the Shannon–Boltzmann–Gibbs entropic form, is always super-additive for any bipartite statistically independent distributions. In this paper, we show that when imposing a suitable constraint, there exist classes of maximal entropy distributions labeled by a positive real number ℵ>0 that makes Kaniadakis entropy multi-additive, i.e., $S_{\kappa }\left[p^{A\cup B}\right]=\left(1+ℵ\right)\;\left(S_{\kappa }\left[p^{A}\right]+S_{\kappa }\left[p^{B}\right]\right)$, under the composition of two statistically independent and identically distributed distributions $p^{A\cup B}\left(x,\;y\right)=p^{A}\left(x\right)\;p^{B}\left(y\right)$, with reduced distributions $p^{A}\left(x\right)$ and $p^{B}\left(y\right)$ belonging to the same class.

## 1. Introduction

A possible generalization of conventional statistics, named κ-statistics, is founded on Kaniadakis entropy (κ-entropy) [1,2,3]. This is a continuous one-parameter deformation of the information functional, also known as the Shannon–Boltzmann–Gibbs (SBG) entropic form, defined in

(1) $$\begin{array}{c} S_{\kappa }\left[p\right]=-\int_{D}p\left(x\right)\;\ln_{\kappa }\left(p(x)\right)\;dx, \end{array}$$

in the appropriate dimensionless unities, where D is a suitable integration domain and

(2) $$\begin{array}{c} \ln_{\kappa }\left(x\right)=\frac{x^{\kappa }-x^{-\kappa }}{2\;\kappa }, \end{array}$$

is a deformed version of the standard logarithm that, in the κ→0 limit, reduces to the ordinary logarithm: $\ln_{0}\left(x\right)\equiv \ln\left(x\right)$. Is then clear that, in the same limit, entropy $S_{\kappa }$ reproduces also the standard expression of SBG-entropy.

Over the last 15 years, the statistics theory based on the κ-entropy has attracted the interest of many researchers, who have studied its foundations on the physical ground [4,5,6,7,8,9,10,11] and its mathematical aspects [12,13,14,15,16,17]. Concurrently, κ-statistic has been employed in various fields. A non-exhaustive list of applications of κ-entropy and κ-distribution includes, to cite a few, those in thermodynamics [18,19]; plasma physics and astrophysics [20,21,22,23,24,25,26]; nuclear physics [27,28,29,30]; cosmological issues [31,32,33], including dark energy [34,35,36] and holographic theory [37,38,39]; information theory [40,41,42,43,44,45,46]; genomics [47,48]; complex networks [49,50]; the economy [51,52,53]; and finance [54,55,56].

As is known [57], for a joined statistical system described by the bipartite probability distribution $p^{A\cup B}\left(x,\;y\right)$ of two statistically independent distributions $p^{A}\left(x\right)$ and $p^{B}\left(y\right)$, i.e., $p^{A\cup B}\left(x,\;y\right)=p^{A}\left(x\right)\;p^{B}\left(y\right)$, the κ-entropy $S_{\kappa }\left[p^{A\cup B}\right]$ is a super-additive quantity, being

(3) $$\begin{array}{c} S_{\kappa }\left[p^{A\cup B}\right]>S_{\kappa }\left[p^{A}\right]+S_{\kappa }\left[p^{B}\right]. \end{array}$$

The difference between the total entropy of a joined system A∪B, and the sum of the entropies of the single parts A and B is sometimes called the *entropic excess*. This is defined in

(4) $$\begin{array}{c} S^{\mathrm{exc}}=S\left[p^{A\cup B}\right]-\left(S\left[p^{A}\right]+S\left[p^{B}\right]\right), \end{array}$$

where, depending on the entropy nature, it can be a positive or negative quantity. It quantifies the information gain or loss in a bipartite system, a property that may be related to the concept of super-stability or sub-stability in thermodynamics [58]. Entropy is super-additive, and the systems it describes are thermodynamically super-stable if the entropic excess is positive. On the other hand, entropy is sub-additive, and the systems it describes are thermodynamically sub-stable if the entropic excess is negative.

Following [59], in agreement with the second principle of thermodynamics, super-stable systems (with a positive entropic excess) tend to join together while sub-stable systems (with a negative entropic excess) tend to fragment.

The opposite of the entropic excess is called the *entropic defect*. Recently, the entropic defect has been investigated as a basic concept of thermodynamics which is able to characterize the entropic form describing a given physical system [60,61].

As discussed in [9], the entropic excess of the κ-entropy for any pair of statistically independent distributions is always positive (cf. Equation (3)), indicating that, in this case, it is a super-additive quantity useful for characterizing super-stable systems.

In general, the entropic excess depends on the bipartite distribution $p^{A\cup B}$, and cannot be quantified in a precise manner. In this work, we show that, within κ-statistics, there are classes of maximal entropy probability distributions, labeled by a real positive parameter ℵ>0, such that the entropic excess of a statistically independent bipartite system is proportional to the sum of the entropy of the single distributions. Thus, for any pair of probability distribution functions (pdf) belonging to the same ℵ-class, we have

(5) $$\begin{array}{c} S_{\kappa }^{\mathrm{exc}}=ℵ\;\left(S_{\kappa }\left[p^{A}\right]+S_{\kappa }\left[p^{B}\right]\right), \end{array}$$

so that the joint κ-entropy $S_{\kappa }\left[p^{A\cup B}\right]$ turns out to be related directly to the sum of the κ-entropies of the single distributions, according to the relation

(6) $$\begin{array}{c} S_{\kappa }\left[p^{A\cup B}\right]=\left(1+ℵ\right)\;\left(S_{\kappa }\left[p^{A}\right]+S_{\kappa }\left[p^{B}\right]\right). \end{array}$$

We call this propriety *multi-additivity* of κ-entropy.

The structure of this paper is as follows. In Section 2, we present the mathematical background related to κ-statistics and the proprieties of composability of κ-entropy for a bipartite statistically independent system. Section 3 contains our main results. There, we introduce the multi-additivity of κ-entropy, and investigate the variational problem concerning the maximization of the κ-entropy under the usual constraints given by the distribution momenta and the multi-additivity conditions. In Section 4, we show by a numerical evaluation that the problem admits solutions at least within the family of Gibbs-like distributions. Finally, Section 5 contains our conclusive comments.

## 2. Mathematical Background

To start with, let us consider the following functional-differential equation [1]:

(7) $$\frac{d}{d\;x}\left[x\;\Lambda (x)\right]=\lambda \;\Lambda \left(\frac{x}{\alpha }\right),$$

where λ and α are two scaling parameters to be determined. It is easy to verify that a solution of Equation (7), with the boundary conditions Λ(1)=0 and $\left(d/d\;x\right)\;\Lambda \left(x\right){|}_{x=1}=1$, is given by the κ-logarithm (2), provide the scaling constants are set as

(8) $$\begin{array}{ccc} \lambda & = & \sqrt{1-\kappa^{2}}, \end{array}$$

(9) $$\begin{array}{ccc} \alpha & = & {\left(\frac{1-\kappa }{1+\kappa }\right)}^{\frac{1}{2\;\kappa }}. \end{array}$$

As will be clarified in a follow-up, the constant λ plays the role of the scaling factor in the argument of pdf, while the constant α is a κ-deformed version of the reciprocal Neperian number. In the κ→0 limit, λ→1 and α→1/e.

The κ-logarithm, defined in ${ℜ}^{+}\to ℜ$, is symmetric in κ→−κ, with $\ln_{\kappa }\left(1\right)=0$, $\lim_{x\to +\infty }$$\ln_{\kappa }\left(x\right)\to +\infty$, as well as $\lim_{x\to 0}\ln_{\kappa }\left(x\right)\to -\infty$. Furthermore, it is a continuous, strictly increasing $\left(d/dx\right)\ln_{\kappa }\left(x\right)>0$ and concave $\left(d^{2}/dx^{2}\right)\ln_{\kappa }\left(x\right)>0$ function for |κ|<1. More importantly,

(10) $$\begin{array}{c} \ln_{\kappa }\left(\frac{1}{x}\right)=-\ln_{\kappa }\left(x\right), \end{array}$$

is a well-known propriety of standard logarithm that is preserved in its κ-deformed version. Finally, since it is in the κ→0 limit, κ-logarithm collapses to the standard logarithm function; this legitimates us considering the κ-logarithm a *faithful generalization of the logarithmic function*.

As the κ-logarithm is a monotonic function, its inverse, the κ-exponential, surely exists, and is given in

(11) $$\begin{array}{c} \exp_{\kappa }\left(x\right)={\left(\kappa \;x+\sqrt{1+\kappa^{2}\;x^{2}}\right)}^{\frac{1}{\kappa }}. \end{array}$$

It is a function defined in $ℜ\to {ℜ}^{+}$, symmetric in κ→−κ, reduces to the standard exponential in the κ→0 limit, and, like the standard exponential, is a continuous, strictly increasing $\left(d/dx\right)\exp_{\kappa }\left(x\right)>0$ and convex $\left(d^{2}/dx^{2}\right)\exp_{\kappa }\left(x\right)<0$ function, with $\exp_{\kappa }\left(0\right)=1$, $\lim_{x\to -\infty }\exp_{\kappa }\left(x\right)\to 0$ as well as $\lim_{x\to +\infty }\exp_{\kappa }\left(x\right)\to +\infty$.

Again, the well-known propriety of the standard exponential is also satisfied by its deformed version

(12) $$\begin{array}{c} \exp_{\kappa }\left(-x\right)=\frac{1}{\exp_{\kappa }\left(x\right)}, \end{array}$$

and, therefore, the κ-exponential is a *faithful generalization of the exponential function*.

Another solution of Equation (7) with the same scaling constants (8) and (9), but with different boundary conditions Λ(1)=1 and $\left(d/d\;x\right)\Lambda \left(x\right){|}_{x=1}=0$, is given by [62]

(13) $$u_{\kappa }\left(x\right)=\frac{x^{\kappa }+x^{-\kappa }}{2},$$

which is a function defined in ${ℜ}^{+}\to {ℜ}^{+}$, and is symmetric in κ→−κ, with $\lim_{x\to 0}u_{\kappa }\left(x\right)=\lim_{x\to +\infty }u_{\kappa }\left(x\right)\to +\infty$. Furthermore, $u_{\kappa }\left(x\right)$ is a continuous, concave $\left(d^{2}/dx^{2}\right)u_{\kappa }\left(x\right)>0$ function for |κ|<1, and obtains its minimum at x=1, where $u_{\kappa }\left(1\right)=1$. Finally, $u_{\kappa }\left(x\right)$ satisfies a dual relation of (10); that is,

(14) $$\begin{array}{c} u_{\kappa }\left(\frac{1}{x}\right)=u_{\kappa }\left(x\right), \end{array}$$

while, in the κ→0 limits, it becomes merely a constant, $u_{0}\left(x\right)=1$. Therefore, there is not an equivalent function in the standard, undeformed formalism.

En passant, we observe that the two scaling constants α and λ are related by the relations

(15) $$\begin{array}{c} -\lambda \;\ln_{\kappa }\left(\alpha \right)=\lambda \;u_{\kappa }\left(\alpha \right)=1. \end{array}$$

One might easily persuade oneself that the functions $u_{\kappa }\left(x\right)$ and $\ln_{\kappa }\left(x\right)$ are strongly connected. In fact, two equivalent analytical expressions of these two functions are given by

(16) $$\begin{array}{ccc} & & \ln_{\kappa }\left(x\right)=\frac{1}{\kappa }\;\sinh\left(\kappa \;\ln(x)\right), \end{array}$$

(17) $$\begin{array}{ccc} & & u_{\kappa }\left(x\right)=\cosh\left(\kappa \;\ln(x)\right), \end{array}$$

which show their relationship with the trigonometric hyperbolic functions. Consequently, many properties of $\ln_{\kappa }\left(x\right)$ and $u_{\kappa }\left(x\right)$ follow from the corresponding properties of sinh(x) and cosh(x). In particular, it is ready to verify that

(18) $$\begin{array}{ccc} & & \ln_{\kappa }\left(x\;y\right)=\ln_{\kappa }\left(x\right)\;u_{\kappa }\left(y\right)+u_{\kappa }\left(x\right)\;\ln_{\kappa }\left(y\right), \end{array}$$

(19) $$\begin{array}{ccc} & & u_{\kappa }\left(x\;y\right)=u_{\kappa }\left(x\right)\;u_{\kappa }\left(y\right)+\kappa^{2}\;\ln_{\kappa }\left(x\right)\;\ln_{\kappa }\left(y\right). \end{array}$$

as a consequence of the additivity formulas of hyperbolic functions.

In addition, it is useful to recall the following relations relating these two functions

(20) $$\begin{array}{c} u_{\kappa }\left(x\right)=\sqrt{1+\kappa^{2}\;\ln_{\kappa }^{2}\left(x\right)}=\ln_{\kappa }\left(x\right)-\lambda \;\ln_{\kappa }\left(\alpha \;x\right), \end{array}$$

that become trivial relations (1=1) in the κ→0 limit, since, in the same limit, λ→1 and $\alpha \to e^{-1}$.

By using the first of these relations, Equation (18) can be rewritten in

(21) $$\begin{array}{c} \ln_{\kappa }\left(x\;y\right)=\ln_{\kappa }\left(x\right)\;\sqrt{1+\kappa^{2}\;\ln_{\kappa }^{2}\left(y\right)}+\ln_{\kappa }\left(y\right)\;\sqrt{1+\kappa^{2}\;\ln_{\kappa }^{2}\left(x\right)}, \end{array}$$

which implies the relevant inequality

(22) $$\begin{array}{c} \ln_{\kappa }\left(xy\right)<\ln_{\kappa }\left(x\right)+\ln_{\kappa }\left(y\right), \end{array}$$

holding in the statistically meaningful interval 0<x,y<1.

The next step is to introduce the κ-entropy $S_{\kappa }\left[p\right]$. Like in the standard case, where SBG-entropy is defined as the negative of the linear average of the Hartley function (or surprise function) defined by $h\left(p\right)=\ln\left(p(x)\right)$, it is natural to introduce the κ-entropy as the negative of the linear average of the κ-deformed Hartley function, $h_{\kappa }\left(p\right)=\ln_{\kappa }\left(p(x)\right)$; that is,

(23) $$\begin{array}{c} S_{\kappa }\left[p\right]=-\langle h_{\kappa }\left(p\right)\rangle , \end{array}$$

a relation that reproduces Equation (1), accounting for the usual definition of the linear average of a statistical observable O(x), given by

(24) $$\begin{array}{c} \langle O\rangle =\int_{D}O\left(x\right)\;p\left(x\right)\;dx. \end{array}$$

It is natural, in analogy with definition (23), to introduce the auxiliary function $I_{\kappa }\left[p\right]$, as the linear average of $u_{\kappa }\left(p(x)\right)$, according to the relation

(25) $$\begin{array}{c} I_{\kappa }\left[p\right]=\langle u_{\kappa }\left(p\right)\rangle . \end{array}$$

This quantity is a positive definite, with $I_{\kappa }\left[p\right]>1$ for any normalized pdf and, in the κ→0 limit, it gives

(26) $$\begin{array}{c} \lim_{\kappa \to 0}I_{\kappa }\left[p\right]=\int_{D}p\left(x\right)\;dx=1. \end{array}$$

Therefore, there is no equivalent function in standard statistics. However, like $\ln_{\kappa }\left(x\right)$ and $u_{\kappa }\left(x\right)$, that are two strictly related functions, also $S_{\kappa }\left[p\right]$ and $I_{\kappa }\left[p\right]$ turn out to be recurrent in the developing of the κ-statistics.

In particular, by taking the linear average of Equations (18) and (19) for a statistically independent bipartite distribution, with $p^{A\cup B}\left(x,\;y\right)=p^{A}\left(x\right)\;p^{B}\left(y\right)$, we obtain

(27) $$\begin{array}{ccc} & & S_{\kappa }\left[p^{A\cup B}\right]=I_{\kappa }\left[p^{A}\right]\;S_{\kappa }\left[p^{B}\right]+S_{\kappa }\left[p^{A}\right]\;I_{\kappa }\left[p^{B}\right], \end{array}$$

(28) $$\begin{array}{ccc} & & I_{\kappa }\left[p^{A\cup B}\right]=I_{\kappa }\left[p^{A}\right]\;I_{\kappa }\left[p^{B}\right]+\kappa^{2}\;S_{\kappa }\left[p^{A}\right]\;S_{\kappa }\left[p^{B}\right], \end{array}$$

stating the additivity rule of $S_{\kappa }$ and $I_{\kappa }$ for two statistically independent systems.

From Equation (27), we readily deduce the super-additive propriety of κ-entropy summarized in Equation (3). In particular, in the κ→0 limit, according to (26), we recover the usual additivity rule of the SBG entropy while, in the same limit, Equation (28) reduces to a trivial identity.

As discussed in [9], composition rule (27) can be rewritten also by means of κ-parentropy, a quantity defined as

(29) $$\begin{array}{c} S_{\kappa }^{*}\left[p\right]=-\lambda \;S_{\kappa }\left[\alpha \;p\right]-1, \end{array}$$

which is a scaled version of κ-entropy.

In fact, by taking the average of Equation (20), we can obtain the following relationship

(30) $$\begin{array}{c} I_{\kappa }\left[p\right]=1+S_{\kappa }^{*}\left[p\right]-S_{\kappa }\left[p\right], \end{array}$$

that relates κ-entropy and κ-parentropy to the $I_{\kappa }$ function. In this way, Equation (27) can be rewritten in

(31) $$\begin{array}{ccc} S_{\kappa }\left[p^{A\cup B}\right] & = & S_{\kappa }\left[p^{A}\right]+S_{\kappa }\left[p^{B}\right]-2\;S_{\kappa }\left[p^{A}\right]\;S_{\kappa }\left[p^{B}\right]\\ & & +S_{\kappa }\left[p^{A}\right]\;S_{\kappa }^{*}[p^{B}]+S_{\kappa }\left[p^{B}\right]\;S_{\kappa }^{*}[p^{A}], \end{array}$$

providing a composition rule for $S_{\kappa }$ that formally only includes κ-entropy; however, it is important to remark that $S_{\kappa }$ and $S_{\kappa }^{*}$ are, actually, two independent quantities.

From Equation (31), the entropic excess of the κ-entropy is given by

(32) $$\begin{array}{c} S_{\kappa }^{\mathrm{exc}}=S_{\kappa }\left[p^{A}\right]\;S_{\kappa }^{*}[p^{B}]+S_{\kappa }\left[p^{B}\right]\;S_{\kappa }^{*}[p^{A}]-2\;S_{\kappa }\left[p^{A}\right]\;S_{\kappa }\left[p^{B}\right]\geq 0, \end{array}$$

that is a quantity defined only as a function of the κ-entropy.

Finally, let us note that Equations (27) and (28) can be combined to write the κ-entropy of a statistically independent multi-partite system in terms of $S_{\kappa }$ and $I_{\kappa }$ of a single distribution. For instance, given a statistically independent tri-partite system, we can obtain the relation

(33) $$\begin{array}{ccc} S_{\kappa }\left[p^{A\cup B\cup C}\right] & = & \kappa^{2}\;S_{\kappa }\left[p^{A}\right]\;S_{\kappa }\left[p^{B}\right]\;S_{\kappa }\left[p^{C}\right]+S_{\kappa }\left[p^{A}\right]\;I_{\kappa }\left[p^{B}\right]\;I_{\kappa }\left[p^{C}\right]\\ & & +I_{\kappa }\left[p^{A}\right]\;S_{\kappa }\left[p^{B}\right]\;I_{\kappa }\left[p^{C}\right]+I_{\kappa }\left[p^{A}\right]\;I_{\kappa }\left[p^{B}\right]\;S_{\kappa }\left[p^{C}\right], \end{array}$$

and so on.

## 3. Multi-Additivity in Kaniadakis Entropy

Within κ-statistics, a maximal entropy pdf may be derived by maximizing the κ-entropy under certain appropriate boundary conditions. Quite often, they are given using linear averages of certain functions $O_{i}\left(x\right)$ as

(34) $$\begin{array}{c} \langle O_{i}\rangle =\int_{D}O_{i}\left(x\right)\;p\left(x\right)\;dx, \end{array}$$

where i=0,1,…,M, with M+1 being the number of given constraints. These relations fix the values of different quantities $\langle O_{i}\rangle$, related to the system under inspection, whose spectra of possible outcomes are given by $O_{i}\left(x\right)$. Many times, constraints are given by the momenta of a certain order *n*; that is, $O_{n}\left(x\right)=x^{n}$. For instance, for n=0, we pose $O_{0}\left(x\right)=1$ with $\langle O_{0}\rangle =1$, which fixes the normalization of the distribution; for n=1, we have $O_{1}\left(x\right)=x$ with $\langle O_{1}\rangle \equiv \langle x\rangle$, which fixes the mean value of the distribution; for n=2, we have $O_{2}\left(x\right)=x^{2}$ with $\langle O_{2}\rangle \equiv \langle x^{2}\rangle$, which is related to the variance of the distribution, etc.

In this case, the maximal entropy distribution can be derived from the following variational problem:

(35) $$\begin{array}{c} \frac{\delta }{\delta p(y)}\left(S_{\kappa }\left[p\right]-\sum_{i=0}^{M}\mu_{i}\int_{D}O_{i}\left(x\right)\;p\left(x\right)\;dx\right)=0, \end{array}$$

where $\mu_{i}$ are Lagrange multipliers related to the M+1 constraints.

By accounting for Equation (7) and definition (1), we obtain the maximal entropy pdf in the form

(36) $$\begin{array}{c} p\left(x\right)=\alpha \;\exp_{\kappa }\left(-\frac{1}{\lambda }\sum_{i=0}^{M}\mu_{i}\;O_{i}\left(x\right)\right), \end{array}$$

where the Lagrange multipliers $\mu_{i}\left(\langle O_{0}\rangle ,\;\langle O_{1}\rangle ,\ldots ,\langle O_{M}\rangle \right)$ are fixed throughout Equation (34) and are finally functions of the boundary conditions $\langle O_{i}\rangle$.

It is worthwhile to observe that, given the analytical expression of $\exp_{\kappa }\left(x\right)$ given in (11), Distribution (36) has an asymptotic power-law behavior, i.e.,

(37) $$\begin{array}{c} p\left(x\right)\approx |\kappa \;x^{n}{|}^{1/\kappa }, \end{array}$$

for large *x*, where *n* is the order of the maximal momenta. This fact justifies the κ-statistic in the study of those anomalous systems, often complex systems, characterized by pdfs with heavy tails.

In the following, let us generalize the optimal problem described above to the case in which, in addition to relations (34), we have further constraints that are functions of the pdf itself. In particular, we seek a class of distributions, labeled by a real constant ℵ, maximizing the κ-entropy under the further constraint given by

(38) $$\begin{array}{c} I_{\kappa }\left[p\right]=1+ℵ,\;\forall p\left(x\right)\in ℵ-\mathrm{class}. \end{array}$$

As will be shown in the next section, this class always existed whenever ℵ≥0, at least for the Gibbs-like distributions, provided the constraint $\langle O_{1}\rangle \equiv \langle x\rangle$ falls in a given region fixed by ℵ.

Therefore, we can state the following: *for a bipartite probability distribution function $p^{A\cup B}\left(x,\;y\right)=p^{A}\left(x\right)\;p^{B}\left(y\right)$ of two statistically independent and identically distributed pdfs $p^{A}\left(x\right)$ and $p^{B}\left(y\right)$, belonging to the same ℵ-class that maximizing the κ entropy under the constraint (38), we have*

(39) $$\begin{array}{c} S_{\kappa }\left[p^{A\cup B}\right]=\left(1+ℵ\right)\;\left(\;S_{\kappa }\left[p^{A}\right]+S_{\kappa }\left[p^{B}\right]\right). \end{array}$$

We call this property *multi-additivity*, and we say that κ-entropy is (1+ℵ)-additive whenever relation (39) holds.

We observe that condition ℵ>0 is fixed by the super-additive character of κ-entropy, while the condition ℵ=0 admits only trivial solutions. In fact, as it is straightforward to verify, the two relations

(40) $$\begin{array}{ccc} & & \int_{D}p\left(x\right)\;dx=1, \end{array}$$

(41) $$\begin{array}{ccc} & & \int_{D}\frac{p{\left(x\right)}^{1+\kappa }+p{\left(x\right)}^{1-\kappa }}{2}\;dx=1, \end{array}$$

are consistent only in the trivial case of κ=0 or for an exact distribution p(x)=δ(x).

To derive the pdf maximizing the κ-entropy under the constraints (34) and (38), we pose

(42) $$\begin{array}{c} \frac{\delta }{\delta p(x)}\left(S_{\kappa }\left[p\right]-\nu \;I_{\kappa }\left[p\right]-\sum_{i=0}^{M}\mu_{i}\int_{D}O_{i}\left(x\right)\;p\left(x\right)\;dx\right)=0, \end{array}$$

where ν is the Lagrange multiplier related to Equation (38).

We obtain

(43) $$\begin{array}{c} -\frac{1}{2\;\kappa }\left[\left(1+\kappa \right)\;\left(1+\kappa \;\nu \right)\;p{\left(x\right)}^{\kappa }-\left(1-\kappa \right)\;\left(1-\kappa \;\nu \right)\;p{\left(x\right)}^{-\kappa }\right]-\sum_{i=0}^{M}\mu_{i}\;O_{i}\left(x\right)=0, \end{array}$$

and pose

(44) $$\begin{array}{c} \left\{\begin{array}{c} \left(1+\kappa \right)\;\left(1+\kappa \;\nu \right)=\lambda \left(\nu \right)\;\alpha {\left(\nu \right)}^{-\kappa }\\ \left(1-\kappa \right)\;\left(1-\kappa \;\nu \right)=\lambda \left(\nu \right)\;\alpha {\left(\nu \right)}^{\kappa } \end{array}\right. \end{array}$$

From Equation (43), we obtain

(45) $$\begin{array}{c} -\lambda \left(\nu \right)\ln_{\kappa }\left(\frac{p(x)}{\alpha (\nu )}\right)-\sum_{i=0}^{M}\mu_{i}\;O_{i}\left(x\right)=0, \end{array}$$

that, solved for p(x), gives the pdf in the form

(46) $$\begin{array}{c} p\left(x\right)=\alpha \left(\nu \right)\;\exp_{\kappa }\left(-\frac{1}{\lambda (\nu )}\sum_{i=0}^{M}\mu_{i}\;O_{i}\left(x\right)\right). \end{array}$$

Although this distribution has the same structure as Equation (36), it differs from (36) in that the two functions λ(ν) and α(ν), given by

(47) $$\begin{array}{ccc} & & \lambda \left(\nu \right)=\lambda \;\sqrt{1-\kappa^{2}\;\nu^{2}}, \end{array}$$

(48) $$\begin{array}{ccc} & & \alpha \left(\nu \right)=\alpha \;{\left(\frac{1-\kappa \;\nu }{1+\kappa \;\nu }\right)}^{\frac{1}{2\;\kappa }}, \end{array}$$

now depend on the Lagrange multiplier ν. They fulfill the relations

(49) $$\begin{array}{ccc} \lambda \left(\nu \right)\;\ln_{\kappa }\left(\alpha (\nu )\right) & = & -(1+\nu ), \end{array}$$

(50) $$\begin{array}{ccc} \lambda \left(\nu \right)\;u_{\kappa }\left(\alpha (\nu )\right) & = & 1+\kappa \;\nu , \end{array}$$

that reduce to (15) for ν=0.

The reality condition of distribution (46), which is still symmetric in κ→−κ, requires

(51) $$\begin{array}{c} \left\{\begin{array}{c} \kappa \in (0,\;1/\nu )\cup (1,\;+\infty )\;\mathrm{for}\;\nu >1,\\ \kappa \in (0,\;1)\cup (1/\nu \;+\infty )\;\mathrm{for}\;\nu <1, \end{array}\right. \end{array}$$

while functions λ(ν) and α(ν) reduce to the constants (8) and (9), respectively, in the ν→0 limit. Further, in the same limit, Problem (42) collapses into Problem (35).

Finally, plugging distribution (46) into Equations (34) and (38), they fix the Lagrange multipliers ν and $\mu_{i}$ as functions of boundary conditions $\langle O_{i}\rangle$, that is, $\nu \equiv \nu (ℵ,\;\langle O_{1}\rangle ,\;\ldots ,\;\langle O_{M}\rangle )$ and $\mu_{i}\equiv \mu_{i}\left(ℵ,\;\langle O_{1},\rangle \;\ldots ,\;\langle O_{M}\rangle \right)$, so that the problem is solved definitively.

It is remarkable to note that, when accounting for the normalization of pdf, from Equation (46), we have

(52) $$\begin{array}{c} \alpha {\left(\nu \right)}^{-1}=\int_{D}\exp_{\kappa }\left(-\frac{1}{\lambda (\nu )}\sum_{i=0}^{M}\mu_{i}\;O_{i}\left(x\right)\right)\;dx, \end{array}$$

a relation that suggests the role of α(ν) as a partition function, i.e., $Z\equiv \alpha {\left(\nu \right)}^{-1}$, in the present formalism. However, a word of caution is in order. As is well-known in standard statistics, the partition function accounted for the normalization. Thus, it is related to the corresponding Lagrange multiplier γ by the relation ln(Z)=1+γ. This is not the case for pdf (46), since α(ν) is related to the Lagrange multiplier of constraint (41), whereas Normalization (40) is controlled by the Lagrange multiplier $\mu_{0}$.

To convince yourself of this, it is sufficient to consider the κ→0 limit. In this case, both Constraints (40) and (41) assume the same form, since $I_{0}\equiv \int p\left(x\right)\;dx$, and

(53) $$\begin{array}{c} \lim_{\kappa \to 0}\alpha \left(\nu \right)\to \exp\left(-1-\nu \right), \end{array}$$

is a constant. Therefore, in this limit, Distribution (46) becomes

(54) $$\begin{array}{ccc} \lim_{\kappa \to 0}p\left(x\right) & = & \exp\left(-1-\nu \right)\;\exp\left(-\sum_{i=0}^{M}\mu_{i}\;O_{i}\left(x\right)\right)\\ & = & \exp\left(-1-\nu -\mu_{0}\right)\;\exp\left(-\sum_{i=1}^{M}\mu_{i}\;O_{i}\left(x\right)\right)\\ & = & \frac{1}{Z}\;\exp\left(-\sum_{i=1}^{M}\mu_{i}\;Oi_{i}\left(x\right)\right), \end{array}$$

where, with $\gamma =\nu +\mu_{0}$, we recover the usual definition of the partition function given above.

Finally, we remark that when the distribution has Expression (46), according to Constraint (38) and by using Equations (49) and (50), we obtain

(55) $$\begin{array}{c} \left(1+\kappa^{2}\;\nu \right)\;\langle \sqrt{\lambda {\left(\nu \right)}^{2}+\kappa^{2}\;{\left(\sum_{i=0}^{M}\mu_{i}\;O_{i}\left(x\right)\right)}^{2}}\rangle -\kappa^{2}\;\left(1+\nu \right)\;\sum_{i=0}^{M}\mu_{i}\;\langle O_{i}\rangle =\left(1+ℵ\right)\;\lambda {\left(\nu \right)}^{2}, \end{array}$$

which is a consistent relationship between the Lagrange multipliers and the expectation values of the present statistical model.

## 4. A Numerical Example: The Gibbs-Like Distribution

To show the existence of solutions to the problem under investigation, let us consider the simplest case of a problem with M=2. Thus, we seek a family of pdf maximizing the κ-entropy under the following constraints:

(56) $$\begin{array}{ccc} & & \int_{0}^{\infty }p\left(x\right)\;dx=1, \end{array}$$

(57) $$\begin{array}{ccc} & & \int_{0}^{\infty }x\;p\left(x\right)\;dx=\langle x\rangle , \end{array}$$

(58) $$\begin{array}{ccc} & & \int_{0}^{\infty }\frac{p{\left(x\right)}^{1+\kappa }+p{\left(x\right)}^{1-\kappa }}{2}\;dx=1+ℵ, \end{array}$$

corresponding, respectively, to the normalization, the linear average, and the multi-additivity constraints.

Solving the variational problem (43) in the present case, we obtain the optimizing pdf in the form

(59) $$\begin{array}{c} p\left(x\right)=\alpha \left(\nu \right)\;\exp_{\kappa }\left(-\frac{1}{\lambda (\nu )}\left(\mu_{0}+\mu_{1}\;x\right)\right). \end{array}$$

This is a Gibbs-like distribution since, in the κ→0 limit, standard Gibbs-distribution $p\left(x\right)=\exp\left(-1-\nu -\mu_{0}-\mu_{1}\;x\right)$ is obtained. Otherwise, (59) is a pdf with an asymptotic power-law heavy tail, being $p\left(x\right)\approx {\left(\kappa \;x\right)}^{1/\kappa }$ for κx≫1.

By plugging distribution (59) into Equations (56)–(58), we obtain the system of equations

(60) $$\begin{array}{ccc} I_{1}\left(z\right) & = & \frac{\mu_{1}}{\alpha (\nu )\;\lambda (\nu )}, \end{array}$$

(61) $$\begin{array}{ccc} \frac{\mu_{0}}{\lambda (\nu )}\;I_{1}\left(z\right)+I_{2}\left(z\right) & = & \frac{\mu_{1}^{2}\;\langle x\rangle }{\alpha \left(\nu \right)\;\lambda {\left(\nu \right)}^{2}}, \end{array}$$

(62) $$\begin{array}{ccc} \alpha {\left(\nu \right)}^{\kappa }\;I_{3}\left(z\right)+\alpha {\left(\nu \right)}^{-\kappa }\;I_{4}\left(z\right) & = & 2\;\left(1+ℵ\right)\;I_{1}\left(z\right), \end{array}$$

where $I_{i}\left(z\right)$, i=1,…,4, are elementary integrals given by

(63) $$\begin{array}{ccc} & & I_{1}\left(z\right)=\int_{0}^{z}\frac{x^{\kappa }+x^{-\kappa }}{2}\;dx=\frac{z}{2}\;\left(\frac{z^{\kappa }}{1+\kappa }+\frac{z^{-\kappa }}{1-\kappa }\right), \end{array}$$

(64) $$\begin{array}{ccc} & & I_{2}\left(z\right)=\int_{0}^{z}\frac{x^{2\;\kappa }+x^{-2\;\kappa }}{4\;\kappa }\;dx=\frac{z}{4\;\kappa }\;\left(\frac{z^{2\;\kappa }}{1+2\;\kappa }-\frac{z^{-2\;\kappa }}{1-2\;\kappa }\right), \end{array}$$

(65) $$\begin{array}{ccc} & & I_{3}\left(z\right)=\int_{0}^{z}\frac{x^{2\;\kappa }+1}{2}\;dx=\frac{z}{2}\;\left(\frac{z^{2\;\kappa }}{1+2\;\kappa }+1\right), \end{array}$$

(66) $$\begin{array}{ccc} & & I_{4}\left(z\right)=\int_{0}^{z}\frac{x^{-2\;\kappa }+1}{2}\;dx=\frac{z}{2}\;\left(\frac{z^{-2\;\kappa }}{1-2\;\kappa }+1\right), \end{array}$$

as functions of the quantity

(67) $$\begin{array}{c} z=\exp_{\kappa }\left(-\frac{\gamma }{\lambda (\nu )}\right). \end{array}$$

The system of Equations (60)–(62) can be solved numerically to obtain the Lagrange multipliers $\mu_{0}\left(ℵ,\;\langle x\rangle \right),\;\mu_{1}\left(ℵ,\;\langle x\rangle \right)$ and ν(ℵ,〈x〉) as functions of the constraints ℵ and 〈x〉.

**Figure 1:**
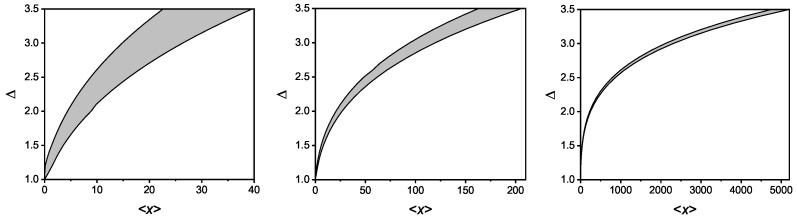
In the figure, we plotted the region of real solutions of system (60)–(62) in the plane of constraints, for several values of the deformation parameter κ. The shaded areas represent the admissible domine.

For any fixed value of ℵ, real solutions only exist in certain intervals of 〈x〉. This is shown in Figure 1, where the region of existence of real solutions (shaded areas) of the system of Equations (60)–(62) is depicted for several values of the deformation parameter κ. The case κ=0 (not reported in the figure) corresponds to the horizontal line passing for ℵ=0. In this case, κ-entropy becomes 1-additive for any pdf.

In other words, each value of ℵ selects a class of distributions whose interval $\left({\langle x\rangle }_{\min},\;{\langle x\rangle }_{\max}\right)$ determines the possible pdf for which the κ-entropy is (1+ℵ)-additive.

In Table 1, we give some numerical values of the interval $\left({\langle x\rangle }_{\min},\;{\langle x\rangle }_{\max}\right)$ for the ℵ-classes between 0.5 and 2.5, step 0.5, corresponding to the three values of the deformation parameter κ reported in the figure.

As an example, let us consider the case with κ=0.3 and ℵ=1.0. Any pair of κ-deformed Gibbs-like distributions with 20.59<〈x〉<26.70 is 2-additive; that is, $S_{\kappa }\left(p^{A}\;p^{B}\right)=2\;\left(S_{\kappa }\left(p^{A}\right)+S_{\kappa }\left(p^{B}\right)\right)$. For instance, take $\langle x_{A}\rangle =22.5$ and $\langle x_{B}\rangle =25.5$; we can evaluate the numerical values of the Lagrange multipliers corresponding to constraints (56)–(58).

These can be read from Table 2, where we show several numerical values of the Lagrange multipliers $\mu_{0},\;\mu_{1}$ and ν, obtained from the system of Equations (60)–(62), for several values of constraints ℵ and 〈x〉 belonging to the allowed region, and corresponding to the three values of the deformation parameter κ reported in the figure.

From this table, we can obtain the terna of multiplier values (−2.1989,0.01849,−0.8219), corresponding to the distribution $p^{A}\left(22.5\right)$, and (−7.3990,0.009404,−1.0796), corresponding to the two distributions $p^{B}\left(25.5\right)$. Then, the respective values of κ-entropy of these two distributions $p^{A}$ and $p^{B}$ are readily evaluated in $S_{\kappa }\left(p^{A}\right)=5.63627$ and $S_{\kappa }\left(p^{B}\right)=5.70258$, while the value of κ-entropy for the join system $S_{\kappa }(p^{A\cup B})=22.6777$, which is exactly the attended result.

## 5. Conclusions

In this work, we showed that, within κ-statistics, there exist classes of pdf that maximize κ-entropy under the condition of constant $I_{\kappa }\left[p\right]$, a problem that admits a solution at least for the family of Gibbs-like distributions. In this way, for any pair of distributions belonging to the same ℵ-class, fixed by the real number ℵ>0, κ-entropy turns out to be (1+ℵ)-additive; that is, the value of κ-entropy of a bipartite statistically independent distribution, whose reduced belonging to the same ℵ-class is a multiple of the sum of the single κ-entropy according to Equation (39).

Equivalently, for any pair of distributions belonging to the same ℵ-class, the entropic excess is proportional to the sum of the κ-entropy of the single pdfs, according to (5).

On the physical ground, Distribution (46) describes a statistical ensemble constrained by condition (38). While the physical meaning of functional $I_{\kappa }$ is still unclear, it seems to be related to the κ-partition function and, consequently, to the κ-free energy of the system, as discussed in [57] (see also [45]). This also applies to Distribution (59), which characterizes a canonical ensemble that is further constrained by (38). Moreover, given two independent physical systems, both members of the same ℵ-class but with different internal energy, the joined κ-entropy is an ℵ-multiple of the sum of their respective κ-entropies. This propriety could be useful for studying thermal and mechanical equilibrium, where the composability of entropy plays a role [62]. However, the potential impact that multi-additivity might have on this aspect of the κ-thermostatistic deserves further investigation.

Furthermore, looking at Equations (27) and (30), we see that the entropic excess in κ-statistics is related to the difference between the κ-entropy and κ-parentropy. In the κ=0 case (standard statistics), such a difference is always null (ℵ=0), i.e., parentropy and entropy have a constant gap equal to 1 for any pdf. Otherwise, when κ>0, the difference between the κ-entropy and κ-parentropy depends on pdf. As shown in this paper, there exist classes of distributions that optimize the κ-entropy under constraint (38), such that the difference between the κ-entropy and κ-parentropy is fixed and equal to ℵ for any pdf belonging to the same ℵ-class.

In other words, the difference between distribution (36) and distribution (46) can be stated as follows: the former assigns distinct values for $S_{\kappa }$ and $I_{\kappa }$, as these functionals both depend on the expectation values $\langle O_{i}\rangle$. In contrast, the latter assigns distinct values for $S_{\kappa }$, but assumes a constant value for $I_{\kappa }=1+ℵ$, fixed prior, for any distribution that falls within the same ℵ-class. In this way, the entropic excess turns out to be proportional to the sum of the κ-entropies of the two systems that are members of the same ℵ-class.

## Figures and Tables

**Table 1:** Permitted interval $\left({\langle x\rangle }_{\min},\;{\langle x\rangle }_{\max}\right)$ for the ℵ-classes between 0.5 and 2.5, step 0.5, corresponding to the three values of the deformation parameter κ reported in the figure.

	κ=0.2		κ=0.3		κ=0.4	
ℵ	${\langle x\rangle }_{\min}$	${\langle x\rangle }_{\max}$	${\langle x\rangle }_{\min}$	${\langle x\rangle }_{\max}$	${\langle x\rangle }_{\min}$	${\langle x\rangle }_{\max}$
0.5	36.8	41.3	5.85	8.06	1.34	3.57
1.0	224.0	246.0	20.59	26.70	4.36	8.87
1.5	787.0	863.0	48.53	61.90	8.78	16.09
2.0	2107.0	2306.0	94.40	119.90	14.81	26.36
2.5	4752.0	5193.0	163.10	206.10	22.64	39.63

**Table 2:** Several numerical values of the Lagrange multipliers, $\mu_{0},\;\mu_{1}$ and ν, obtained from the system (60)–(62), for some values of constraints ℵ and 〈x〉 in the allowed region, corresponding to the three values of the deformation parameter κ reported in the figure.

	κ=0.2				κ=0.3				κ=0.4			
ℵ	〈x〉	$\mu_{0}$	$\mu_{1}$	ν	〈x〉	$\mu_{0}$	$\mu_{1}$	ν	〈x〉	$\mu_{0}$	$\mu_{1}$	ν
	37	2.5761	0.03342	2.0607	6.0	0.8102	0.1980	0.5634	1.5	0.1690	0.8377	0.08488
0.5	38	−1.3791	0.01671	−0.8298	6.5	−1.7817	0.09376	−0.9642	2.0	−2.1727	0.3281	−1.1016
	39	−3.6921	0.01204	−1.5989	7.0	−3.5685	0.06270	1.3475	2.5	−3.9538	0.1719	−1.2830
	40	−6.7617	0.009670	−1.9744	7.5	−6.1733	0.04751	−1.5050	3.0	−6835	0.1113	−1.2834
	225	1.2720	0.003917	0.8586	21.0	0.3309	0.04680	0.2129	5.0	−1.4060	0.1438	−0.6946
1	230	−1.8947	0.001918	−0.8003	22.5	−2.1989	0.01849	−0.8219	6.0	0.0608	−0.9088	−0.9088
	235	−4.1393	0.001391	−1.2201	24.0	−4.1330	0.01231	−1.0079	7.0	−5.0451	0.03775	−0.9106
	240	−7.2656	0.001124	−1.4248	25.5	−7.3990	0.009404	−1.0796	8.0	−8.6768	0.02712	−0.8879
	790	2.2376	0.001219	1.4587	50.0	−0.5497	0.01190	−0.2549	9.0	0.1923	0.1678	0.1149
1.5	810	−2.1479	0.0004171	−0.6953	52.5	−2.2195	0.006305	−0.6605	11.0	−2.7096	0.03009	−0.7113
	830	−4.9360	0.0002896	−1.0202	55.0	−3.6418	0.004508	−0.7731	13.0	−4.6906	0.01655	−0.7141
	850	−10.1219	0.0002300	−1.1654	57.5	−5.4875	0.003571	−0.8236	15.0	−8.3442	0.01136	−0.6919
	2150	−1.3309	0.0001541	−0.4175	95	1.1453	0.01147	0.6736	16.0	−1.2338	0.03603	−0.4690
2	2200	−3.9385	0.00009907	−0.7820	100	−1.7453	0.003185	−0.4927	19.0	−3.1742	0.01251	−0.5956
	2250	−7.4910	0.00007668	−0.9237	105	−3.2500	0.002083	−0.6232	22.0	−5.1612	0.007630	−0.5848
	2300	−26.3122	0.00006387	−1.0015	110	−5.0400	0.001591	−0.6727	25.0	−9.1792	0.005478	−0.5660
	4800	0.08936	0.00008495	0.03164	170	−1.2742	0.001911	−0.3547	23.0	0.1565	0.05803	0.08391
2.5	4900	−2.6290	0.00004530	−0.5566	180	−3.1060	0.001066	−0.5263	28.0	−2.9118	0.007853	−0.508
	5000	−5.0810	0.00003349	−0.7245	190	−5.2257	0.0007732	−0.5753	33.0	−5.0884	0.004362	−0.4981
	5100	−9.2667	0.00002734	−0.8089	200	−9.9583	0.0006167	−0.5964	38.0	−9.8852	0.003019	−0.4796

## Data Availability

No new data were created or analyzed in this study. Data sharing is not applicable to this article.
